# Case Report: H3K27M-Mutant Glioblastoma Simultaneously Present in the Brain and Long-Segment Spinal Cord Accompanied by Acute Pulmonary Embolism

**DOI:** 10.3389/fonc.2021.763854

**Published:** 2022-02-08

**Authors:** Buajieerguli Maimaiti, Salamaitiguli Mijiti, Ting Jiang, Yinyin Xie, Weixuan Zhao, Yu Cheng, Hongmei Meng

**Affiliations:** Department of Neurology and Neuroscience Center, First Hospital of Jilin University, Changchun, China

**Keywords:** H3K27M-mutant glioblastoma, brain glioblastoma, multicentric glioblastoma, spinal glioblastoma, pulmonary embolism, case report

## Abstract

**Background:**

Glioblastoma multiforme (GBM) is a highly malignant glioma that rarely presents as an infratentorial tumor. Multicentric (MC) gliomas involve lesions widely separated in space or time, and MC gliomas involving supra- and infratentorial brain regions are rare. In most cases, the infratentorial lesion is seen after surgical manipulation or radiation therapy; it is typically located in the cerebellum or the cervical region, manifesting as metastasis originating from the brain. Besides, venous thromboembolism in brain tumors is usually seen after craniotomy.

**Case Presentation:**

We present an uncommon adult case of symptomatic H3K27M-mutant MC glioblastoma simultaneously present in the brain, fourth ventricle, and cervical and lumbar spinal cord regions accompanied by acute pulmonary artery embolism in an adult woman who had not undergone previous therapeutic interventions. We also review the literature on this interesting presentation.

**Conclusion:**

Our report highlights that clinicians should be alert to the potential alarming presentation of GBM. The incidence of spinal metastasis of cerebral GBM is increasing. Patients with a prior diagnosis of GBM with or without any new onset in the spinal cord should undergo an early MRI of the spinal cord to confirm the diagnosis at an early stage. While management of GBM remains controversial, more research is needed to explore molecular features of GBM further and develop novel targeted therapies for these patients.

## 1 Introduction

Most glioblastoma multiforme (GBMs) occur in the supra-tentorium, including the frontal, temporal, parietal, and occipital lobes. Only a very small proportion of GBMs occur in other areas of the central nervous system (CNS), such as the cerebellum, brain stem, and spinal cord (1). The H3K27M mutation can be frequently found in pediatric diffuse midline glioma (DMG) and is associated with a more aggressive clinical course. However, this mutation is very rarely found in adults. The H3K27M mutation is observed throughout the CNS’s midline structures, mainly in the thalamus, brain stem, and spinal cord (2). H3K27M-mutant tumors are designated as World Health Organization grade IV tumors, regardless of their histological features (3, 4). Multicentric (MC) glioblastoma with many infratentorial lesions mainly occurs in patients who have had prior surgical treatment or radiotherapy (5, 6). Although related cases or case series have been reported previously, intracranial H3K27M-mutant GBM with spinal lesions before chemoradiotherapy or craniotomy is not common.

This paper presents an uncommon adult case of a patient presenting with back pain, headache and vomiting, inability to walk, and incontinence when first admitted to our hospital. Related findings revealed an H3K27M-mutant MC glioblastoma simultaneously present in the brain, periventricular area, and cervical, thoracic, and lumbar spinal cord at the time of initial diagnosis. During hospitalization, the patient also had an acute pulmonary embolism (PE), presenting with respiratory symptoms, including breathing difficulty and severe right-sided anterior and posterior chest pain. We also review the literature on this uncommon presentation.

## 2 Case Description

### 2.1 Patient Demographics, Chief Complaint, and History

A 61-year-old woman presented to our department with a 3-week history of progressive bilateral weakness of her lower limbs. She had severe walking impairment with associated dizziness, headache, nausea, and vomiting. Although in good spirits, she reported anorexia, insomnia, and urinary and fecal incontinence, with a recent 5-kg weight loss. The patient denied a history of hypertensive disease, diabetes mellitus, and infectious diseases, such as hepatitis and tuberculosis. She also denied a history of respiratory symptoms such as fever, dyspnea, cough, and sputum. She had no food or drug allergies and no familial genetic disorders. She denied tobacco or alcohol use and had no history of surgery or trauma.

### 2.2 Physical Examination

On the first admission, the patient’s vital signs were stable, body temperature was normal (37.0°C), blood pressure was 153/92 mmHg, heart rate was 86 beats per minute, and the breath sounds and respiratory rate were normal. Her speech was initially fluent. Her pupils were equal in size, 3-mm diameter, and reacted appropriately to light. No apparent ocular muscle weakness was present, although she had horizontal nystagmus. There was no facial muscle weakness, and her tongue extension was centered. The muscle strength of both upper limbs was normal. Kernig’s sign was negative.

At the presentation, the examination was notable for movement disorders having grade -1 muscle strength in both her lower limbs, with active tendon reflexes. The swelling was observed in her left lower limb. The Babinski and Chaddock reflexes were positive bilaterally. There was a profound sensory disturbance with hypoesthesia at the T6 level and below with reduced vibration sense. The bilateral finger-nose and rapidly alternating tests were normal when testing for cerebellar dysfunction, but she could not perform the bilateral heel–knee–shin test.

On the 2nd day after admission, the patient developed severe chest pain with intermittent nausea and vomiting, chest tightness, and dyspnea. After the blood test and imaging results were available, she was diagnosed with PE. During hospitalization, about 2 weeks after the admission, the patient developed a progressively worsening speech disturbance. The patient can understand others but is not fluent in her speech. Before discharge, that patient was in poor condition. She was indifferent to answering any questions. Her vital signs and other physical examinations were the same as her admission.

### 2.3 Diagnostic Assessment

#### 2.3.1 Laboratory Tests

The patient tested arterial oxygen saturation at the time of the dyspnea and chest pain, indicating a partial oxygen pressure of 63 mmHg. The D-dimer blood level was elevated, 11.26 μg/ml (normal range: 0–1 μg/ml). The cerebrospinal fluid (CSF) was yellow with elevated pressure, >400 mmH2O. CSF examination showed elevated protein level 30.81 g/l (normal range: 0.15–0.45 g/l), but low levels of glucose, 1.6 mmol/l (normal range: 2.3–4.1 mmol/l), and chloride, 110 mmol/l (normal range: 119–129 mmol/l). Moreover, for the white cell count (WBC-BF: 34.00 × 10^6^/l), 96.0% of the content comprised lymphocytes and 4.0% monocytes; however, the red blood cell count was 0/L.

#### 2.3.2 Imaging Findings

**Figure 1:**
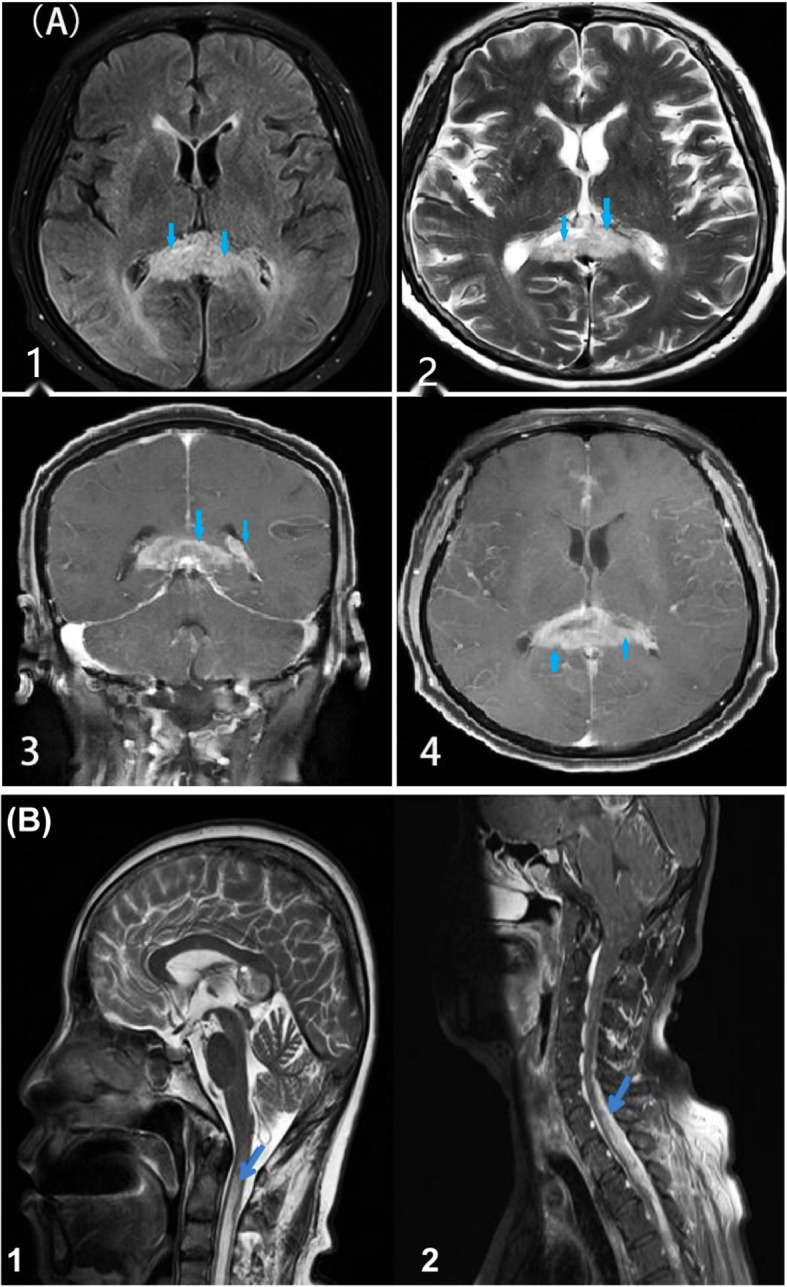
MRI lesions in brain and spinal cord. [**(A)**, 1–4] Head MRI shows lesions (marked with arrows) in the corpus callosum and left lateral periventricular area. T1 [**(A)** 1] shows a slightly low signal, while T2 [**(A)** 2] shows unevenly slightly high and low signals. Enhanced MRI [**(A)** 3–4] shows a high signal mass in the corpus callosum and left lateral periventricular area, with marked marginal enhancement and narrowing of the left lateral ventricle by compression. [**(B)** 1–2] Cervical spine MRI shows high-intensity signaling in the spinal cord at the C2–C7 vertebra levels and abnormal enhancement in the spinal cord at the C5–T4 vertebrae (marked with arrows).

**Figure 2:**
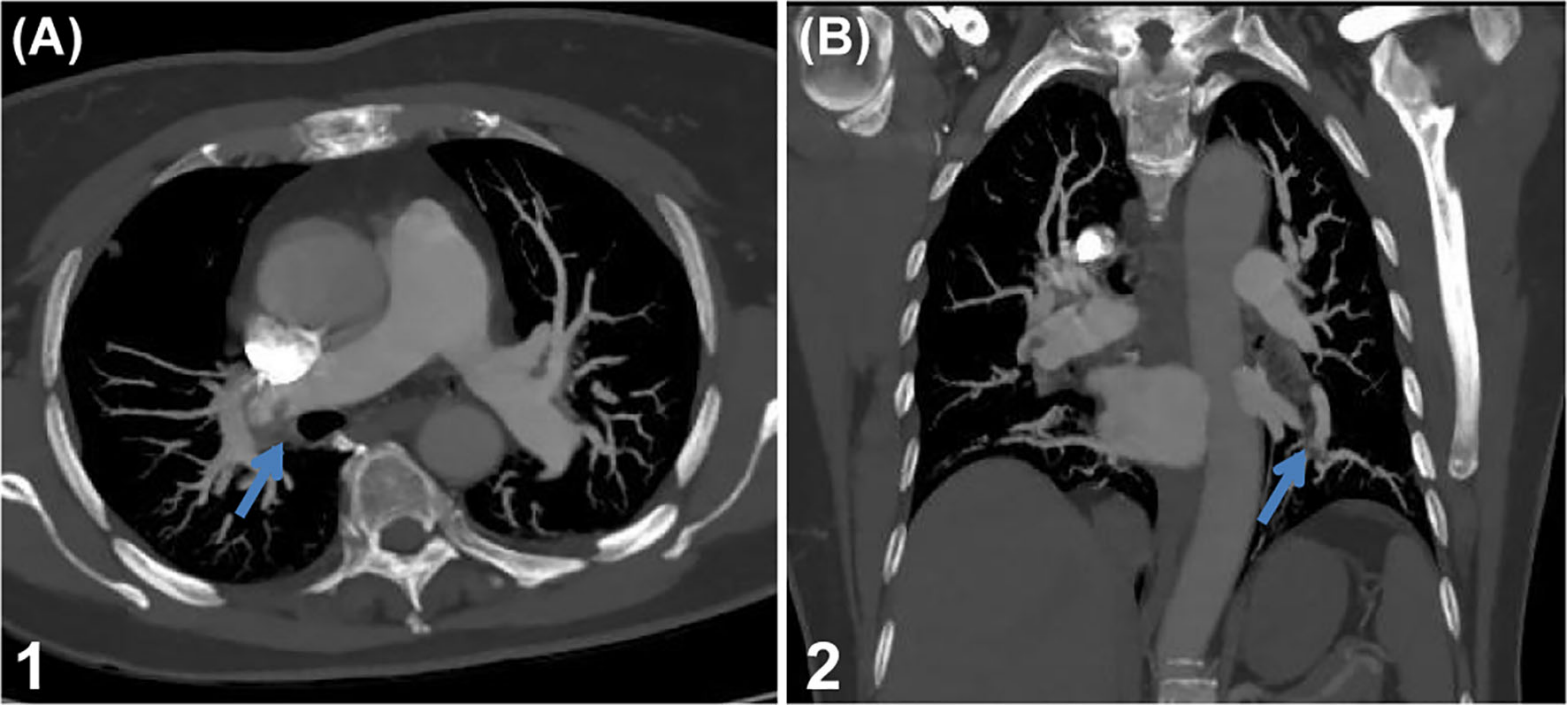
Pulmonary artery embolisms. **(A, B)** Pulmonary computed tomography angiography shows multiple bilateral pulmonary artery embolisms (marked with arrows).

Brain 3T MRI [**Figure 1A** (1–4)] showed lesions in the corpus callosum and the left lateral periventricular area. Combined with the MR spectroscopy results, the lesions were considered tumors. Spectroscopic metabolite analysis at the corpus callosum lesion showed a low N-acetyl aspartate peak and increased choline. Cervical-spine MRI [**Figure 1B** (1–2)] showed high-intensity signaling in the spinal cord at the C2–C7 vertebra levels and abnormal enhancement in the spinal cord at the C5–T4 vertebrae. Positron emission tomography-computed whole-body imaging showed limited hypermetabolic foci in the corpus callosum and diffused metabolic increases in the spinal cord cervicothoracic and lumbosacral regions, which were suggestive of malignancy. No hypermetabolic changes were observed in other organs, including the lungs. Ultrasound scanning of the lower extremities suggested the presence of thrombosis in the left common femoral vein and bilateral intermuscular veins (acute stage, complete type). A pulmonary computed tomography angiogram (**Figures 2A, B**) showed multiple bilateral pulmonary-artery embolisms.

### 2.4 Histopathology Findings

**Figure 3:**
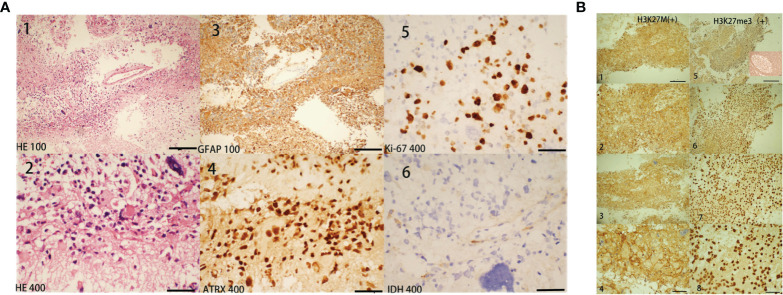
Histopathology findings. Histopathological features of our case diagnosed as H3K27M mutant brain glioblastoma. The biopsy tissues were obtained from the corpus callosum and the lesions shown in brain MRI (in **Figure 1A**, lesions marked with arrows). In **Figure 3**, for ×100 staining the scale bar = 5 µm and for ×400 staining the scale bar = 20 µm. Hematoxylin and eosin (H&E). **Figure 3** [**(A)** 1 and 2] shows dense cell proliferation, with areas of a spindle-cell pattern and some pleomorphism, extensive mitosis, some atypical and karyomegaly, and nuclear irregularity. At approximately 40% [**(A)** 5: Ki67], the cell proliferation rate was high, showing positive glial fibrillary acid protein [**(A)** 3: GFAP]. This neoplastic lesion was positive for *ATRX* [**(A)** 4], *H3K27M* mutation [**(B)** 1-4], and *H3K27me3*(B5-8), while it was negative for *IDH1R132H* mutation [**(A)** 6]. Unfortunately, biopsy tissues of our patient are insufficient for further identification of *H3K27me3*(+) cells and for the squeezing of *H3K27M* and *H3K27me3*. Figure 3B-5 also showed positive H3k27me3 ICH staining of tonsil tissue for comparison.

Biopsy showed that the pathological diagnosis was grade IV glioblastoma with necrosis not otherwise specified. Immunohistochemistry staining (**Figure 3**) results were CD20(-), CD3(-), CD79a (-), PAX-5(-), LCA (-), Ki-67(>40%), GFAP (+), Olig-2(+), ATRX (+), MAP2(+), P53(+30%), *IDH1R132H* mutation (-), MGMT (+30%), MBP (-), NeuN (-), Syn (+), vimentin (+), nestin (+), CD56(+), *H3K27me3*(+), and *H3K27M* mutation (+). According to the 2016 World Health Organization Classification of Tumors of the Central Nervous System, this case lacked molecular test results; there was insufficient evidence to classify this case as a distinct disease entity.

### 2.5 Interventions and Follow-Up Findings

The biopsy tissues were obtained from the corpus callosum, and the lesions are shown in brain MRI (in **Figure 1**, lesions marked with arrows). The family and patient decided that palliative care was the best option for the brain and spinal cord gliomas because she felt severely unwell and had non-operable multicentric midline GBM lesions. The patient developed deep vein thrombosis (VTE) and PE during hospitalization. Therefore, she was administered rivaroxaban 15 mg orally twice daily for the first 3 weeks. After the follow-up, we learned that the patient gradually became unconscious at home, and his respiratory and cardiac functions gradually worsened. The patient died 42 days after hospital discharge. The time from symptom onset to death was approximately 3 months.

## 3 Discussion

### 3.1 Incidence Rate and Risk Factors of GBM

GBM is a highly malignant adult primary brain tumor with the most devastating prognosis among gliomas. Glioblastoma accounts for 14.6% of all primary brain and other CNS tumors, 48.3% of primary malignant brain tumors, and 57.3% of gliomas (1). GBM is mainly diagnosed at older ages with the highest rates in individuals aged 75.0 to 84.0 years and with a median age of diagnosis of 64.0 years (7). In addition, a higher incidence of GBM has been reported in males than in female patients (1). Researchers are uncertain of the possible etiological risk factors for glioma to date. Reported risk factors for GBM are prior therapeutic radiation, immune factors, immune genes, and some single-nucleotide polymorphisms detected by genome-wide association studies (8). There is no current evidence of an association of GBM with lifestyle characteristics such as cigarette smoking, alcohol and drug consumption, dietary exposure to N-nitroso compounds (cured or smoked meat or fish), pesticide exposure, obesity, or head injury (8, 9). Our patient was a 61-year-old Asian woman. She did not present any of the risk factors associated with glioma development.

### 3.2 Clinical Features of Spinal Metastasis of GBM

Intracranial GBM with spinal lesions before chemoradiotherapy or craniotomy is defined as initial spinal metastasis, and its incidence varies from 0.4% to 2.0% (6). Spinal cord GBM generally manifests the following: back pain, radicular pain, paresis, sensory loss, urinary retention, or incontinence (10). Spinal metastases of GBM can be detected using spinal MRI (6). Our patient initially presented with spinal cord symptoms (including back pain, paresis, incontinence, and loss of sensation) and gradually presented aggravated intracranial symptoms (including nausea, vomiting, dizziness, and a progressive speech disorder). Brain MRI of our patient [(**Figure 1A** (1–4)] showed lesions in the corpus callosum and left lateral periventricular area. Cervical-spine MRI [**Figure 1B** (1–2)] showed high-intensity signaling in the spinal cord at the C2–C7 vertebrae and abnormal enhancement in the spinal cord at the C5–T4 vertebrae. At the same time, enhanced thickening of the meninges was observed.

A systematic review among 86 patients identified from 51 published articles; the mean age of intracranial GBM patients with spinal metastases was 46.8 years, and among them, 59.7% were male. Moreover, the most common symptom was lumbago or cervicalgia (90.2%), followed by paraparesis (86.0%) and bowel/bladder dysfunction (approximately 80.0%). Out of those 86 cases, only 22 patients were from Asia (11). A recently published study showed a higher incidence (13.0%) of initial spinal metastasis of GBM and suggested the importance of spinal screening using MRI in patients with or without spinal symptoms. This study also concluded that intracranial GBM with initial spinal lesions presented higher incidences of intracranial dissemination and was located at subventricular zones (10). However, we immediately performed brain and spinal cord MRI, resulting in a speedy diagnosis. Some MC spinal metastases of intracranial GBM have been documented in previous reports up to 2019 (6, 11). Data from those reviews suggest that any new onset of back pain or neurological deficit of the extremities in patients with a prior diagnosis of GBM should indicate suspected spinal metastasis. Besides, in younger and healthier GBM patients, the possibility of developing spinal metastases is higher than in elderly GBM patients, and this is most probably due to a more prolonged overall survival in the younger and healthier population (12, 13). A case report demonstrated FDG PET/CT appearances of GBM recurrence with diffuse spinal cord metastasis (12).

### 3.3 Spreading Mechanisms for Spinal Metastasis of GBM

Spreading mechanisms of GBM to the spinal cord mainly include spreading by contiguity along compact fiber pathways and through CSF following exfoliation of tumor cells (12). Invasion of a primary tumor into the cortical surface can also lead to subpial spread, followed by leptomeningeal spread (6). Therefore, the CSF study is essential for the early diagnosis of GBM patients who developed atypical symptoms during treatment or in the follow-up period (13). In this case, although the spinal cord symptoms occurred before the brain symptoms, we believe that the lesion in the corpus callosum was the primary lesion, and the lesions in the spinal cord were dropped metastasis from the brain *via* the leptomeningeal spread. Nevertheless, we found no malignant cells in the CSF. The leukocyte CSF count was high, and lymphocytes accounted for 96% of these cells, suggesting viral infection. However, we did not identify any virus in the CSF lymphocytes, assuming that the lymphocytes in CSF were likely related to leptomeningeal spread. However, some cases of spinal metastases of intracranial GBM have also been reported with no previous history of cranial or craniospinal radiation (5, 14, 15). The patient in our report also did not undergo previous surgery or radiotherapy. In a report by Wright, the leptomeningeal disease was present in 53.5% of patients, intramedullary disease in 53.2%, and intradural extramedullary disease in 47.1% (11). The postoperative drainage devices also cause the patient to develop spinal metastases with poor clinical outcomes (16).

### 3.4 Histopathological and Genetic Features in GBM

Molecular characteristics for glioma classification are IDH mutation, chromosome 1p/19q deletion, histone mutations, and other genetic parameters such as TRX loss, TP53 and TERT mutations, and DNA methylation levels (17). IDH wild-type tumors are common in older patients with a worse prognosis. In contrast, IDH mutant tumors are common in younger patients with better predictions (9, 18). In our case, despite the absence of genetic test results, immunohistochemistry (IHC) at the molecular level suggested Ki-67 (>40%), IDH1R132H mutation (-), GFAP (+), ATRX (+), MAP2 (+), P53 (+30%), MGMT (+30%), H3K27me3 (+), and H3K27M mutation (+). Characterized by a constant midline location and low rate of MGMT promoter methylation, most H3K27M mutant tumors are aggressive with a poor prognosis, including tumors that demonstrate low-grade histological features. Therefore, these tumors are designated as World Health Organization grade IV tumors, regardless of their histological features (3, 4). This case was diagnosed as an H3K27M-mutant, MC midline GBM (grade IV) involving the brain and extensive spinal cord. In most H3K27M-mutant glioma, H3K27me3-stained cells were reduced, and large areas of staining were missing. *H3K27M* inhibits the activity of polycomb repressive complex 2 by interacting with the methyltransferase *EZH2*, leading to a further decrease in *H3K27me3* content (19). A pathological study of pediatric H3K27M-mutant gliomas using IHC presented that the majority of low-grade gliomas presented with higher rates of positive H2K27me3 staining. Although the mechanism of tumorigenesis in H3K27M-mutant spinal cord gliomas with retained H3K27me3 expression in this study is still unclear, the authors considered that spinal GBM would have biological characteristics similar to these low-grade gliomas. The authors also found that H3K27me3 was lower or absent in tumor cells but retained in endothelial cells and infiltrating lymphocytes (20). However, in our case, *H3K27me3* staining was positive; we considered that *H3K27me3* might be restored in vascular endothelial and infiltrating lymphocytes or tumor cells. Unfortunately, the biopsy tissues of our patient are insufficient for further identification of *H3K27me3*(+) cells and the squeezing of *H3K27M/H3K27me3*.

### 3.5 Therapy and Prognosis of Spinal Metastasis of GBM

There are still no definitive guidelines for treating spinal metastasis from cerebral GBM. Previously reported therapeutic options include surgical decompression, chemotherapy, and radiotherapy of the craniospinal axis (25 to 40 Gy) for spinal metastases of GBM (11). Some authors suggest that treatment for spinal metastases of GBM was only for pain relief and neurologic deficits but exerted no effect on the overall outcome (21, 22). Chemoradiotherapy is the typical treatment method after diagnosis of spinal metastasis. Surgery can be performed for focal lesions, but chemoradiotherapy is the only option for diffuse or leptomeningeal disease (21, 22). The anatomical location and the infiltrative nature of GBM make total resection of the tumor mass virtually impossible (17). Recent studies focused on precision oncology or targeted therapy, nanotechnology, immunotherapy, and a ketogenic diet to develop advanced therapeutic strategies that enable a more comprehensive GBM therapy (17, 23–26).

In H3K27M-mutant midline gliomas (DMG), maximal safe resection is rarely implemented due to the sensitive tumor location, so small partial resection is attempted when feasible (27). Recent work has explored an alternative approach to targeting H3K27M-mutant DMG by applying chimeric antigen receptor (CAR) T cells. In adult GBM, CAR T cells engineered against human epidermal growth factor receptor 2, highly expressed on the surface of a subset of GBM, have shown early anecdotal efficacy (27, 28). Moreover, in another study, the histone mutation *H3K27M* is regarded as a catalyst within neuro-oncology and can be used to develop new therapeutic pathways. Sequencing of ctDNA in CSF allows investigators to detect tumor mutations (29). Detecting ctDNA of *H3K27M* mutant glioma *via* CSF elicits the possibility of leveraging ctDNA as an early biomarker for disease progression in DMG (27). Our patient received only palliative care because of the severity of her overall condition.

With current treatment limitations involving surgery, radiotherapy, and chemotherapy, there is a high rate of treatment failure and recurrence for GBM, leading to a poor prognosis for patients (30). For spinal GBM metastasis, studies reported a poor prognosis with a median survival of 2.0 to 4.0 months only after diagnosis (6). In a systematic review by Wright et al., the mean time between diagnosis of the primary brain GBM and spinal metastasis was 13.5 months. The median time between diagnosis of spinal GBM metastasis and death was 2.8 months (13). However, the molecular features, severity of tumor invasion, presentation, and prognosis in our case were consistent with those of high-grade glioma.

### 3.6 Risk Factors and Mechanisms of VTE in GBM

VTE is a common complication in patients with primary brain tumors. According to a meta-analysis, 20.0% of brain tumors develop VTE yearly (31). VTE includes DVT and PE. Factors associated with increased risk of VTE in patients with GBM have been reported. Tumor-related factors include the GBM subtype (a higher incidence in high-grade tumors), the presence of intra-tumoral thrombosis, the presence of *IDH1* wild-type status, and “‘podoplanin expression (32). Reported treatment-related risk factors are surgery, tumor biopsy subtotal tumor resection, use of corticosteroids, and anti-VEGF therapy. Additionally, laboratory parameters and hemostatic biomarkers such as high white blood cell count, low platelet count, high soluble P-selectin levels, elevated coagulation factor VIII activity, and increased D-dimer levels are also associated VTE occurrence in brain tumors (32). A recently published study concluded that older age, body mass index, preoperative activated partial thromboplastin time, D-dimer, tumor histology, and surgery duration independently increased the risk of postoperative DVT/PE in patients with brain tumors (33).

Multiple mechanisms, including vascular abnormalities, overexpression of tissue factor, and release of procoagulant microparticles (extracellular vesicles) by tumor cells, have been proposed for the occurrence of thrombosis in GBM (34). Relating these mechanisms to clinical presentations of thrombosis can lead us to a more causality-based, personalized, and possibly cancer-specific thromboprophylaxis and treatment. However, during hospitalization, our patient was a 61-year-old woman who developed VTE, including DVT and PE. When patients with spinal cord metastasis present with back pain, the same manifestation due to PE is easily overlooked. In this case, we completed a timely pulmonary CTA by testing our patient’s blood D-D and oxygen values and promptly prevented death from acute PE.

## 4 Conclusion

In conclusion, we reported an interesting adult case of H3K27M-mutant MC-GBM that involved the brain and extensive spinal cord regions at the time of initial diagnosis with an acute fatal medical complication, without any previous therapeutic interventions. This report suggests that GBM is a diffuse disease with a devastating prognosis. The occurrence of spinal metastasis of cerebral GBM is increasing due to prolonged overall survival. Patients with a prior diagnosis of GBM with or without any new onset in the spinal cord should undergo an early MRI of the spinal cord. FDG PET/CT and CSF examination can help confirm the diagnosis of spinal metastases at an early stage. Mechanisms related to clinical presentations of thrombosis can lead us to a more causality-based, personalized, and possibly cancer-specific thromboprophylaxis and treatment. While the management of GBM remains controversial, more research is needed to explore molecular features of GBM further and develop novel targeted therapies for these patients.

## Author Contributions

All authors have read and approved the manuscript. The first author, BM, wrote the first draft and was involved in writing and reviewing the completed paper. The corresponding author HM was predominantly engaged in organizing, designing, and writing the article. Other authors contributed equally to the paper’s conception, literature review, writing, and editing of the figures. All authors contributed to the article and approved the submitted version.

## Conflict of Interest

The authors declare that the research was conducted in the absence of any commercial or financial relationships that could be construed as a potential conflict of interest.

## Publisher’s Note

All claims expressed in this article are solely those of the authors and do not necessarily represent those of their affiliated organizations, or those of the publisher, the editors and the reviewers. Any product that may be evaluated in this article, or claim that may be made by its manufacturer, is not guaranteed or endorsed by the publisher.
