# Supplementary figures and images for: Case Report: H3K27M-Mutant Glioblastoma Simultaneously Present in the Brain and Long-Segment Spinal Cord Accompanied by Acute Pulmonary Embolism
Source: Front Oncol. 2022 Feb 8;11:763854. doi: 10.3389/fonc.2021.763854 (PMC8861510; doi:10.3389/fonc.2021.763854)

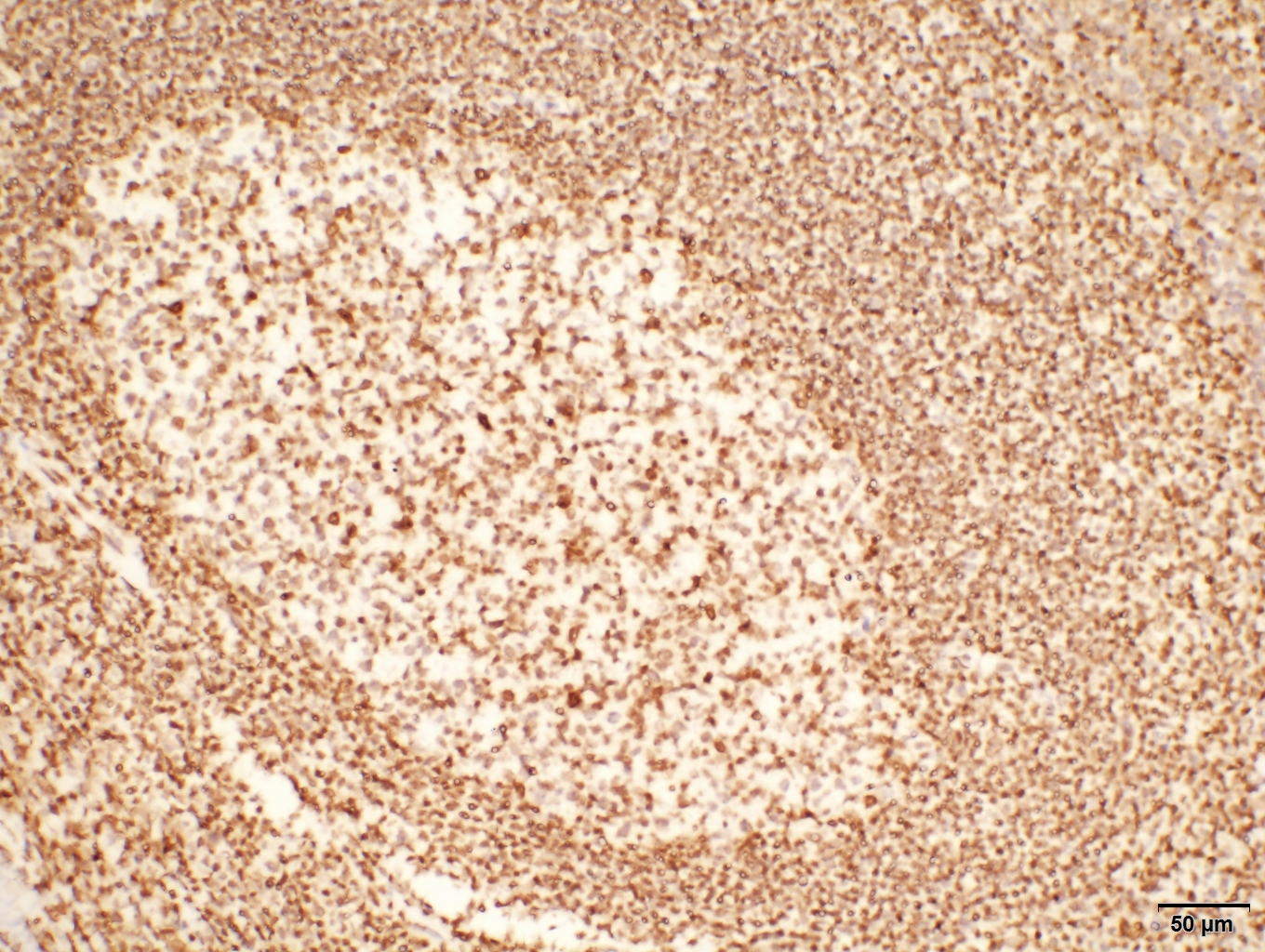

Supplement: Supplementary file 1 [file Image_1.png]
